# Effect of Reduction Methods on the Properties of Composite Films of Bacterial Cellulose-Silver Nanoparticles

**DOI:** 10.3390/polym15142996

**Published:** 2023-07-10

**Authors:** Ratchanon Jenkhongkarn, Muenduen Phisalaphong

**Affiliations:** Bio-Circular-Green-Economy Technology & Engineering Center (BCGeTEC), Department of Chemical Engineering, Faculty of Engineering, Chulalongkorn University, Bangkok 10330, Thailand; ratchanon.je@gmail.com

**Keywords:** bacterial cellulose, silver nanoparticle, biocomposite film

## Abstract

Composite films of bacterial cellulose-silver nanoparticles (BC-Ag) were prepared by different methods of in situ reduction of silver ions, using sodium hydroxide, ascorbic acid, chitosan, and UV irradiation. The effects of the reduction methods on their properties were investigated. The chitosan-reduced composite exhibited dispersed silver nanoparticles (AgNPs) within the nanocellulose matrix with the smallest size, while the ascorbic-reduced composite displayed the largest size. The incorporation of AgNPs tended to reduce the crystallinity of the composites, except for the ascorbic-reduced composite, which exhibited an increase in crystallinity. Mechanical testing revealed that the ascorbic-reduced composite had the highest Young’s modulus of 8960 MPa, whereas the UV-reduced composite had the highest tensile strength and elongation at break. Thermal analysis of BC-Ag composites indicated similar glass transition temperature and decomposition profiles to BC, with additional weight-loss steps at high temperatures. The sodium hydroxide-reduced composite demonstrated the highest electrical conductivity of 1.1 × 10^−7^ S/cm. Water absorption capacity was reduced by the incorporation of AgNPs, except for the chitosan-reduced composite, which showed an enhanced water absorption capacity of 344%. All BC-Ag composites displayed very strong antibacterial activities against *Staphylococcus aureus* and *Escherichia coli.* These results also highlight the potential uses of BC-Ag composites for various applications.

## 1. Introduction

Cellulose is one of the most abundant, readily available, and inexpensive common polymers on earth with an annual production of around 75 billion tons [1]. Cellulose offers significant advantages over petroleum-derived polymers as it is renewable, environmentally friendly, cost-efficient, non-toxic, biodegradable, and biocompatible [2,3,4]. Apart from plants, several microorganisms are known to be producers of cellulose, particularly a gram-negative bacterium called *Gluconacetobacter xylinus*. Bacterial cellulose (BC), produced by bacteria, differs from plant cellulose as it lacks hemicellulose, lignin, and other compounds present in plant cellulose [2], which enables BC to be obtained in higher purity. Moreover, BC consists of an ultrafine and uniform network of cellulose fibers with a diameter ranging from 20 to 100 nm [5]. These characteristics are not observed in plant cellulose, which exhibits a significantly larger fiber diameter (≥10 times larger) [6]. These unique attributes impart BC with remarkable properties, including high purity, crystallinity, and water-holding capacity, alongside excellent mechanical properties, a large surface area, and good biocompatibility and biodegradability [7].

Due to superior properties compared to plant cellulose, BC has found its use in various applications, such as tissue engineering, electronic devices, biomedical applications, and drug delivery systems. In the biomedical field, BC serves as a wound-dressing material, artificial skin, vascular grafts, scaffold for tissue engineering, artificial blood vessel, and medical pads [8,9]. BC has also been utilized as a temporary substitute for burn victims’ skin [10], an environmentally compatible ion exchange membrane in fuel cells [11], and as biocompatible and biodegradable sensors and actuators [12]. However, modifications are necessary to enhance the capabilities and improve the properties of BC materials before they can be effectively utilized in various applications. For instance, pure BC lacks electrical conductivity, magnetism, and hydrophobicity [13]. Therefore, BC cannot be used directly in electrical devices, batteries, sensors, or electrochromic devices [10,13]. Moreover, BC does not possess inherent antimicrobial properties [14], and despite its high mechanical properties, its stress-bearing capacity is impeded due to numerous pores [2]. However, due to its structural characteristics, BC exhibits significant potential as a matrix and reinforcement in composite materials [15]. Recently, different types of BC composites have been synthesized, resulting in enhanced mechanical, biological, and electrical properties [2,16]. Various methods have been employed to improve the properties of BC, including modifications to the synthesis method and culture conditions and the incorporation of BC with other materials to create a different type of BC composite [17,18], which can widen the application of BC in many other areas.

Silver, in the form of nanoparticles (AgNPs) and oxides (mainly Ag_2_O), has been widely used for antibacterial applications. The unique physicochemical properties of nanosilver, including its high surface-to-volume ratio and inherent biocidal activity, make it a promising candidate for combating bacterial infections [19]. However, for practical application, silver needs to be integrated into composites or retained inside a solid support to apply over the affected area, preventing rapid oxidation and increasing its stability [20]. BC contains abundant hydroxyl groups that are capable of reducing Ag^+^ to Ag^0^ [21] and offers an ideal matrix for silver integration due to its porous and interconnected structure. BC could act as both a reducing agent and stabilizing agent in the reduction of silver ions to silver nanoparticles [21]. As a result, there has been significant research dedicated to investigating the incorporation of silver into BC to develop composite materials with enhanced antimicrobial activity and excellent mechanical properties. Several research studies have explored various reduction methods to convert silver ions into silver nanoparticles within the BC matrix. However, previous studies have primarily focused on investigating the antibacterial properties of bacterial cellulose-silver nanoparticles (BC-Ag) composites, neglecting the investigation of other properties. Furthermore, the effects of different reduction methods on the final properties of the composite have rarely been compared.

This research aims to investigate the influences of different methods of in situ reduction of silver ions in a bacterial nanocellulose matrix on the properties of BC-Ag composite films using facile, inexpensive, and easily replicable approaches. The methods applied in this study include chemical reduction using sodium hydroxide (NaOH), ascorbic acid (C_6_H_8_O_6_), and chitosan solutions, and photochemical reduction using UV irradiation. The BC and BC-Ag composites were characterized using various techniques, including X-ray diffraction (XRD), a field-emission scanning electron microscope (FE-SEM), Fourier transform infrared spectra (FT-IR), a universal testing machine (UTM), and electrochemical impedance spectroscopy (EIS), as well as thermal analysis techniques, which include thermogravimetric analysis (TGA) and differential scanning calorimetry (DSC). Additionally, the antibacterial activities of BC-Ag composites were evaluated using the colony-forming count method against gram-positive bacteria (*S. aureus*) and gram-negative bacteria (*E. coli*).

## 2. Materials and Methods

### 2.1. Materials 

The stock culture of *Gluconacetobacter xylinus* bacterial strain AGR 60 was used for BC biosynthesis. Silver nitrate (AgNO_3_) (purity, >99.8%) was purchased from RCI Labscan Ltd. (Bangkok, Thailand). Sucrose and ammonium sulfate were purchased from Ajax Finechem Pty Ltd. (New South Wales, Australia). Acetic acid was purchased from Mallinckrodt Chemicals (Paris, KY, USA). Sodium hydroxide (NaOH) pellets were purchased from Kemaus (New South Wales, Australia). Chitosan from shrimp shells with low molecular weight (∼25 kDa) was purchased from Marine Bio Resources Co., Ltd. (Samutsakhon, Thailand). Ascorbic acid (C_6_H_8_O_6_) was purchased from Loba Chemie Pvt Ltd. (Mumbai, India).

### 2.2. Preparation and Purification of BC

BC was produced through a biosynthesis method utilizing coconut water as the primary ingredient. The culture medium was prepared by combining coconut water with a 1.0% (*v/v)* acetic acid solution (30%, *v/v*), 5.0% (*w/v*) sucrose, and 0.5% (*w/v*) ammonium sulfate). The mixture was then sterilized at 110 °C for 5 min. Pre-cultures were prepared by transferring 15 mL of the *G. xylinus* stock culture to a 500 mL Erlenmeyer flask containing 300 mL of medium. These pre-cultures were statically incubated at 30 °C for 7 days. Subsequently, 5.0% (*v/v*) of the incubated medium was added to 75 mL of the activated medium, which was then placed in a glass Petri dish with a diameter of 14.5 cm and incubated at 30 °C for 7 days.

BC synthesized by the bacteria was harvested at the air-liquid interface of the culture broth after 7 days. Then the harvested materials were purified by rinsing them with deionized water (DI) for 30 min, and then they were immediately placed in 1.0% (*w/v*) NaOH solution at room temperature for 24 h to eliminate any attached media and bacterial cells. Following that, BC was rinsed with running water for 30 min and further washed with deionized water (DI) until the pH became neutral. Afterward, the purified BC pellicle was stored in DI water at 4 °C for subsequent usage.

### 2.3. Impregnation and Reduction of Ag Ions in BC

BC-Ag composite films were prepared using different methods, some of which were modified from the previous reports. For the reduction by NaOH, ascorbic, and UV irradiation, BC pellicles were pretreated by immersing them in 0.02 M AgNO_3_ solution with constant stirring for 2 h and left in the dark for 2 days to ensure complete adsorption of Ag ions into the BC matrix. The reduction of Ag ions by UV irradiation was performed by exposing the BC pellicles to UV light (wavelength of 254 nm, 15 W) for 5 h [22,23]. 

The chemical reduction was conducted using NaOH ascorbic acid, or chitosan. For the reduction of Ag ions using NaOH and ascorbic, the pretreated BC pellicles that had earlier been immersed in the 0.02 M AgNO_3_ solution were rinsed with DI water to eliminate excess AgNO_3_ solution. Subsequently, the pellicles of BC-Ag ions were reduced by immersing them in a solution containing 0.12 M ascorbic acid or 0.12 M NaOH for 30 min [14,24]. For reduction by chitosan, the BC pellicles were pretreated by immersing them in a solution of 0.02 M AgNO_3_, 2.0% (*w/v*) chitosan, and 1.0% (*v/v*) acetic acid, stirring for 2 h, and leaving them in the dark for 2 days. Afterward, the mixture was incubated at 75 ± 2 °C for 5 h. 

After the reduction processes, the pellicles of BC-Ag nanoparticles were then washed with DI water for 10 min to eliminate excess chemicals and then were air-dried at room temperature (30 °C) and stored in plastic film at room temperature. The composite films of BC-Ag nanoparticles prepared by reducing silver ions using UV irradiation, NaOH, ascorbic acid, and chitosan were denoted as BC-Ag(UV), BC-Ag(NaOH), BC-Ag(Ascorbic), and BC-Ag(Chitosan), respectively.

### 2.4. Physical and Chemical Characterization

The functional groups and chemical structure of the BC and BC-Ag composites were determined by Fourier transform infrared (FT-IR) spectroscopy (Spectrum One, Perkin Elmer, MA, USA) in the ranges of 4000–400 cm^−1^ with a resolution of 4 cm^−1^.

The morphologies of the BC and BC composites were observed by scanning with an electron microscope and energy dispersive X-ray spectrometer using a Field Emission Scanning Electron Microscopy (FE-SEM, Thermo Fisher Scientific, Quanta 250 FEG, Hillsboro, OR, USA). The specimens were sputtered with gold. The SEM–EDS was performed at an accelerating voltage of 5–15 kV.

The mechanical properties of dry films of BC and BC-Ag composites, including Young’s modulus, tensile strength, and elongation at break, were assessed following the ASTM D882 (2004) standard using a Universal Testing Machine (Hounsfield H10 KM, Redhill, England). The samples were prepared in the form of rectangular film sheets (1 × 5 cm^2^). To ensure repeatability, a minimum of five specimens for each sample type was tested. The test was performed at a temperature of 25 °C.

The decomposition temperature (T_d_) and residual weight were analyzed using a thermogravimetric analyzer (TGA, NETZSCH TG 209 F3 Tarsus, Germany). Film samples weighing between 3–6 mg were subjected to heating from 30 to 600 °C under a nitrogen atmosphere, with a constant heating rate of 10 °C/min.

The glass transition temperature (T_g_) was determined using a differential scanning calorimeter (DSC, NETZSCH DSC 204 F1 Phoenix, Germany). A total of 7–10 mg of the sample was loaded into an aluminum pan. The heating process took place under a nitrogen atmosphere, ranging from −100 to 300 °C, with a constant heating rate of 10 °C/min.

The crystallinity and structural information of the BC and BC-Ag composites were characterized with an X-ray diffractometer (XRD, Bruker AXS Model D8 Discover, Karlsruhe, Germany) using Cu radiation. The measurements were conducted in the scan range of 2θ from 10–80° with an accelerating voltage of 40 kV and electric current of 40 mA. The crystallinity index (CI) was calculated using DIFFRAC.EVA software (Version 6.0.0.7) with the following formula:

$$\mathrm{Crystallinity}\;\left(\%\right)=\frac{\mathrm{Crystalline}\;\mathrm{area}\times 100}{\mathrm{Total}\;\mathrm{Area}}$$

The electrical properties of BC and BC-Ag composites were determined by Electrochemical Impedance Spectroscopy (EIS, Squidstat Plus, Tempe, AZ, USA) at 25 °C, and Nyquist plots were obtained using the frequency range from 200 kHz to 1 Hz.

The water absorption capacity (WAC) was evaluated by immersing the weighed and dried samples (2 × 2 cm^2^) in DI water at room temperature until they reached equilibrium. The initial weights of the dry samples were recorded as W_d_. After removing the samples from the water and eliminating any excess water on the surface using Kimwipes paper, the weight of each water-swollen sample was then measured, and the weights of the hydrated samples were recorded as W_h_. This process was repeated until no further weight change was observed. The WAC was calculated using the following formula.

$$\mathrm{WAC}\left(\%\right)=\frac{W_{h}-W_{d}}{W_{d}}\times 100$$

### 2.5. Biological Characterization

The modified JIS Z 2801 method was used to evaluate the antimicrobial properties of composite films against *Staphylococcus aureus* and *Escherichia coli*. To begin, a stock cell suspension of *S. aureus* and *E. Coli* was prepared and allowed to incubate at 37 °C for 16–20 h. Next, the samples of composite films (3 × 3 cm^2^) were sterilized with UV light for 2 h, and then 1 mL of the bacterial cell suspension, with an initial cell density of 1.5–4 × 10^6^ CFU/mL, was applied to each sample. Following a 24 h incubation period at 37 °C, the samples were subjected to thorough shaking at 200 rpm for 1 min in the presence of 10 mL of pH 7.4 phosphate-buffered saline (PBS). Subsequently, the PBS solution containing the cells was cultured on agar plates and incubated at 37 °C for another 24 h. Finally, the resulting cell colonies were counted.

## 3. Results and Discussion

### 3.1. Impregnation and In Situ Reduction of Ag Ions

BC is produced from the biosynthesis process using inexpensive coconut water waste as an alternative substrate, which contains nutrient and minerals for microbial growth [25], instead of conventional media like the Hestrin and Schramm (HS) medium. After 7 days, the obtained yield of BC was about 7.0 g dry weight/L. The moisture content of the BC hydrogel was 99.12%. The weight of a 5 × 5 cm^2^ BC hydrogel was 11.02 g (the weight of 5 × 5 cm^2^ dry BC was 0.0964 g). The thickness of the BC hydrogel was approximately 5 mm. To prepare the composites of BC-Ag ions, purified BC pellicles were immersed in AgNO_3_ solution for two days in the dark for the complete adsorption of Ag^+^ into the BC matrix. Abundant hydroxyl groups in the BC provide anchoring sites and react with Ag^+^ [26]. BC could also act as a stabilizing and a capping agent, as well as a template for the synthesis of AgNPs [27], preventing the agglomeration of AgNPs, which typically occurs in a system lacking a capping or stabilizing agent.

After 2 days’ immersion in the AgNO_3_ solution, the BC film appeared slightly darker, while the AgNO_3_ solution remained clear. However, under the reduction with chitosan, the BC pellicles that were immersed in AgNO_3_-chitosan solution became brown and the solution color turned from clear yellow to dark brown. During the incubation at 75 ± 2 °C for 5 h, the colors of the AgNO_3_-chitosan solution and the composite film were gradually darkening over time. In this case, chitosan, which contains an amide group, is able to form an intermolecular bonding with the hydroxyl groups of BC [28] and acts as a constituent material in the final composite. For the reduction using the ascorbic acid and NaOH solution, the AgNO_3_-treated BC film immediately underwent a color change when immersed in the solution of reducing agents. The color of the composite film turned silvery-brown when immersed in the ascorbic acid solution and turned dark brown when immersed in the NaOH solution. Under the reduction using UV light, the composite film gradually turned dark brown when exposed to UV light irradiation. These color changes in the solution and the BC pellicles, consistent with previous cases [22,24], indicate the reduction of Ag ions to AgNPs.

The reduction mechanisms for each method are as follows. In the reduction by ascorbic acid, ascorbic acid serves as a reducing agent by providing electrons to Ag^+^, causing the reduction to Ag^0^ and the formation of dehydroascorbic acid [29]. The reduction mechanism of silver ions by NaOH was proposed by Han et al. [30], who explained that cellulose fiber was capable of reducing Ag^+^ to Ag^0^ at room temperature under a strong alkaline condition. However, Ag^+^ ions were unstable under alkaline conditions, resulting in the rapid formation of insoluble Ag_2_O particles, which could be reduced by the hydroxyl group of the cellulose, creating another pathway for forming silver nanoparticles. In the UV irradiation method, Ag^+^ ions bonded to BC fibers undergo a transformation into silver nanoparticles after being exposed to UV light for a certain period of time, which also results in the formation of Ag_2_O [22]. In the reduction of silver ions by chitosan, the latter could act as both a reducing agent and stabilizing agent in the reduction process of Ag^+^ ions. The reduction ability of chitosan is attributed to the strong coordination abilities and complexation interactions of NH_2_ groups with metal ions [31]. Additionally, in this method, chitosan also serves as a filler within the final composites by penetrating the BC matrix, filling the pores, and forming hydrogen bonds with the BC fibrils during the immersion and reduction process [28].

### 3.2. Morphology

**Figure 1:**
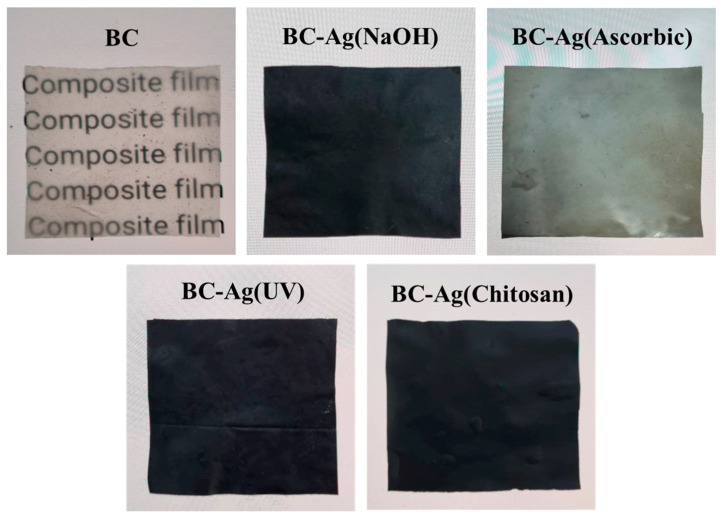
Photographs of dried BC and BC-Ag composites. Transparent and colorless BC films are darkened by the incorporation of Ag. The words “composites film” behind the films illustrate the transparency of each film.

The transparent and colorless BC films are darkened by the incorporation of Ag, as depicted in Figure 1. After drying, the films of BC-Ag(NaOH), BC-Ag(UV), and BC-Ag(Chitosan) turned from dark brown to black, while BC-Ag(Ascorbic) films appeared silver. This disparity in color, with BC-Ag(Ascorbic) appearing silver while the other BC-Ag composites appeared black, could be attributed to the absence of Ag_2_O in BC-Ag(Ascorbic). Ag_2_O has a black or dark brown color, while Ag nanoparticles exhibit various colors depending on their size and shape. All BC-Ag films showed a substantial decrease in opacity compared to transparent BC films, ultimately leading to the complete obstruction of light. The thickness of BC, BC-Ag(NaOH), BC-Ag(Ascorbic), BC-Ag(UV), and BC-Ag(Chitosan) films were measured to be 32 ± 3, 51 ± 4, 34 ± 2, 31 ± 3 and 90 ± 10 µm, respectively. Notably, the BC-Ag(Chitosan) films exhibited a significant increase in thickness, which could be attributed to the diffusion of chitosan into the BC, which formed the intermolecular hydrogen bonds in the BC network [28,32], resulting in an increased thickness of the film. The increased thickness observed in BC-Ag(NaOH) films could be attributed to the integration of nanoparticles, particularly Ag_2_O, in the nanocellulose matrix.

**Figure 2:**
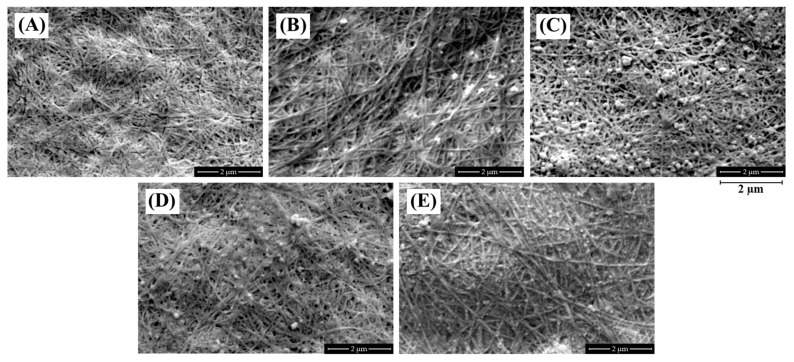
SEM images of dried BC (**A**), BC-Ag(NaOH) (**B**), BC-Ag(Ascorbic) (**C**), BC-Ag(UV) (**D**)**,** and BC-Ag(Chitosan) (**E**) composites at 50,000× magnification.

FE-SEM analysis was performed on dried BC and BC-Ag composite films, with the observed surface morphology at 50,000× magnification presented in Figure 2. The FE-SEM analysis revealed that in all samples, fibers with a consistent diameter can be seen in a mesh-like structure, forming a complex network with different orientations. The fibers exhibit a smooth surface and a cylindrical or ribbon-like shape. The interstitial spaces between the fibers contribute to the porosity of the material, which can be observed as interconnected pores of varying sizes and shapes. This structure helps silver ions diffuse into the BC structure and distribute evenly inside the material and on the fiber surfaces [22].

Silver nanoparticles with varying diameters can also be seen as white dots on the surface of BC-Ag films. The BC-Ag(Ascorbic) film exhibits the largest diameter of AgNPs, while the BC-Ag(Chitosan) film exhibits the smallest diameter. The smaller size of AgNPs in BC-Ag(Chitosan) can be attributed to the stabilizing or capping capabilities of chitosan on metal nanoparticles [31], which prevents AgNPs from agglomerating and helps maintain their smaller sizes. Conversely, the larger size observed in BC-Ag(Ascorbic) could be attributed to the strong reduction ability of ascorbic acid and a lack of stabilizing ability. The size difference of the AgNPs within the BC-Ag composites is of significant importance as it can impact the properties of the materials, such as surface area, reactivity, and their potential applications in fields such as catalysis, sensing, and antimicrobial activities [33,34,35].

**Figure 3:**
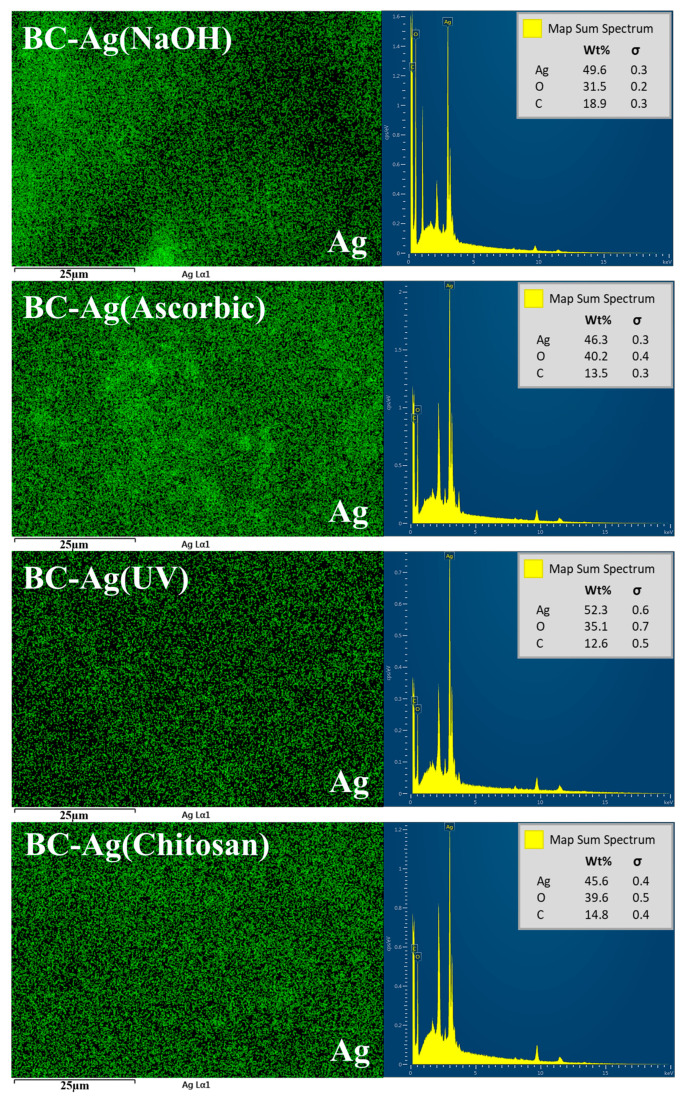
EDX elemental mapping analysis of Ag and map sum spectrum of BC-Ag composites at 5000× magnification.

EDX scanning is also utilized to determine the chemical composition and distribution of Ag on the surface of the BC-Ag composites. Figure 3 presents the elemental mapping analysis of Ag and the EDX quantitative analysis of BC-Ag composites at 5000× magnification. Elemental mapping reveals a uniform distribution of Ag within BC-Ag composites. A prominent peak at around 3 keV from the map sum spectrum analysis confirms the presence of Ag within the materials.

### 3.3. FTIR Analysis

**Figure 4:**
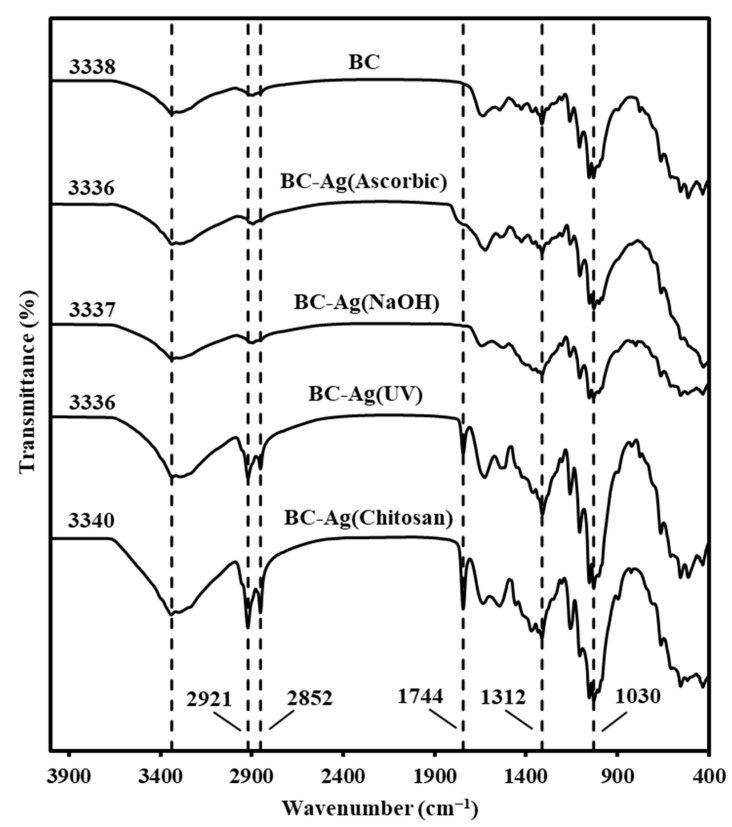
FTIR spectra of BC and BC-Ag composites.

FTIR analysis was performed to investigate the spectra shift and chemical structure. The FTIR spectra of BC and BC-Ag composites are presented in Figure 4. All samples exhibit the characteristic peaks of bacterial cellulose. The first prominent peak is observed as a broad vibration band in the range of 3200–3400 cm^−1^, indicating the stretching vibration of the abundant O-H groups within the BC network [36]. The peak of BC-Ag shifted very slightly from that of BC film located at 3338 cm^−1^, which might be caused by AgNPs interfering with the hydrogen bonding of the BC network. The subsequent peaks observed at approximately 2921 cm^−1^ and 2852 cm^−1^ can be attributed to the asymmetric and symmetric stretching vibration, respectively, of the C-H groups in the cellulose structure [37]. These peaks are most prominent in the BC-Ag(UV) and BC-Ag(Chitosan) composites. Additionally, both composites exhibit a peak at 1744 cm^−1^, which could be assigned to non-conjugated C-C stretching [38]. The presence of these three peaks observed in BC-Ag(UV) and BC-Ag(Chitosan) could indicate the presence of a capping agent with the AgNPs in the BC structure [38]. The intense band between 900–1200 cm^−1^ is attributed to the stretching vibration of the C-O and C-C groups [39]. Furthermore, the band at around 1625 cm^−1^ is assigned to the H-O-H bending of the absorbed water molecules [28], and the band at 1312 cm^−1^ is due to OH in-plane bending [40]. 

### 3.4. Crystallinity

**Figure 5:**
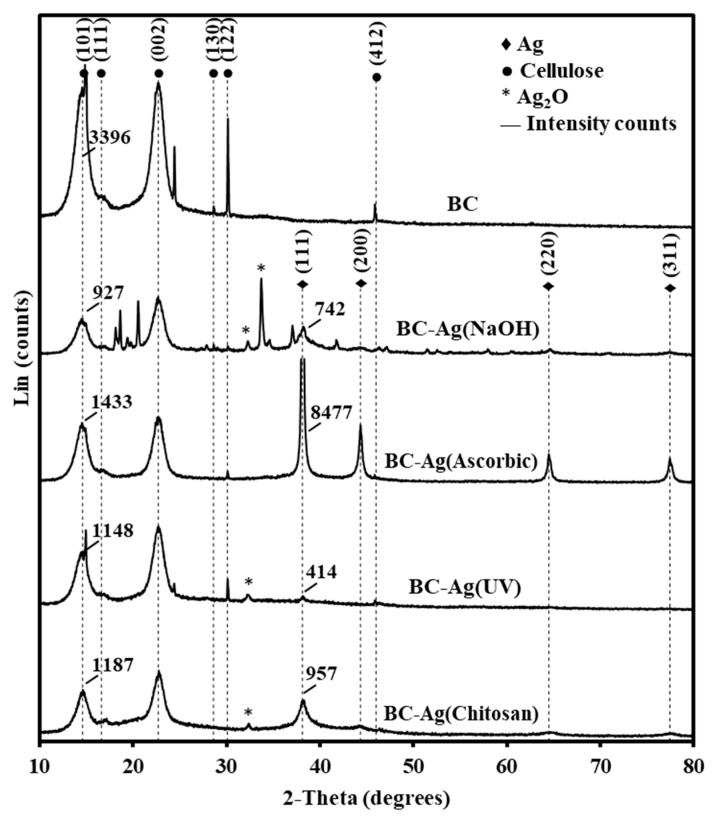
XRD patterns of BC and BC-Ag composites.

The XRD patterns of BC and BC-Ag composites are shown in Figure 5. The XRD diffractograms reveal characteristic peaks of bacterial cellulose in all BC films at 2θ angles of 14.6°, 16.6°, 22.7°, 28.6°, 30.1°, and 46.0°, corresponding to the crystal surface orientations (101), (110), (002), (130), (122), and (412), respectively, belonging to the crystalline structure of cellulose I (JCPDS No.03-0829) [22,37,41]. Additional diffraction peaks at 38.1°, 44.3°. 64.5°, and 77.4° are observed in AgNPs composites, corresponding to the lattice plane values (111), (200), (220), and (311), respectively, of the face-centered cubic metallic-silver crystal (JCPDS No.76-1393) [22]. These peaks indicate the presence of AgNPs within the BC network. The other peaks observed at 32.3° for BC-Ag(NaOH), BC-Ag(UV), and BC-Ag(Chitosan) and at 33.7° for BC-Ag(NaOH) are due to the (111) plane of cubic Ag_2_O and (100) plane of hexagonal Ag_2_O (JCPDS No.72-2108) [42]. Notably, these Ag_2_O peaks are absent in BC-Ag(Ascorbic).

The crystallinity index (CI) was calculated using DIFFRAC.EVA software. The calculated crystallinity index values for BC, BC-Ag(NaOH), BC-Ag(Ascorbic), BC-Ag(UV), and BC-Ag(Chitosan) composites are 78.5%, 68.3%, 83.3%, 69.6%, and 48.6%, respectively. Among the BC-Ag composites, the highest degree of crystallinity was observed in BC-Ag(Ascorbic), while BC-Ag(Chitosan) showed the lowest degree of crystallinity. Except for BC-Ag(Ascorbic), the incorporation of AgNPs resulted in a slight reduction in the crystallinity of the composites, which could be due to silver interfering with hydrogen bonds within the BC matrix [43], while chitosan-reduced composites exhibited a significant reduction of crystallinity. The interaction of chitosan molecules with cellulose fibrils might play a significant role in this effect. Previous studies have suggested that the incorporation of a polymer in the BC network can impact the reflection plane of crystalline cellulose, leading to incomplete orientation and decreasing the composite crystallinity [28,44]. In contrast, the inclusion of AgNPs substantially enhances the crystallinity of the BC-Ag(Ascorbic) composite, possibly due to the interactions between Ag^0^ and BC, as well as the absence of Ag_2_O, which is present in other BC-Ag composites.

### 3.5. Mechanical Properties

**Figure 6:**
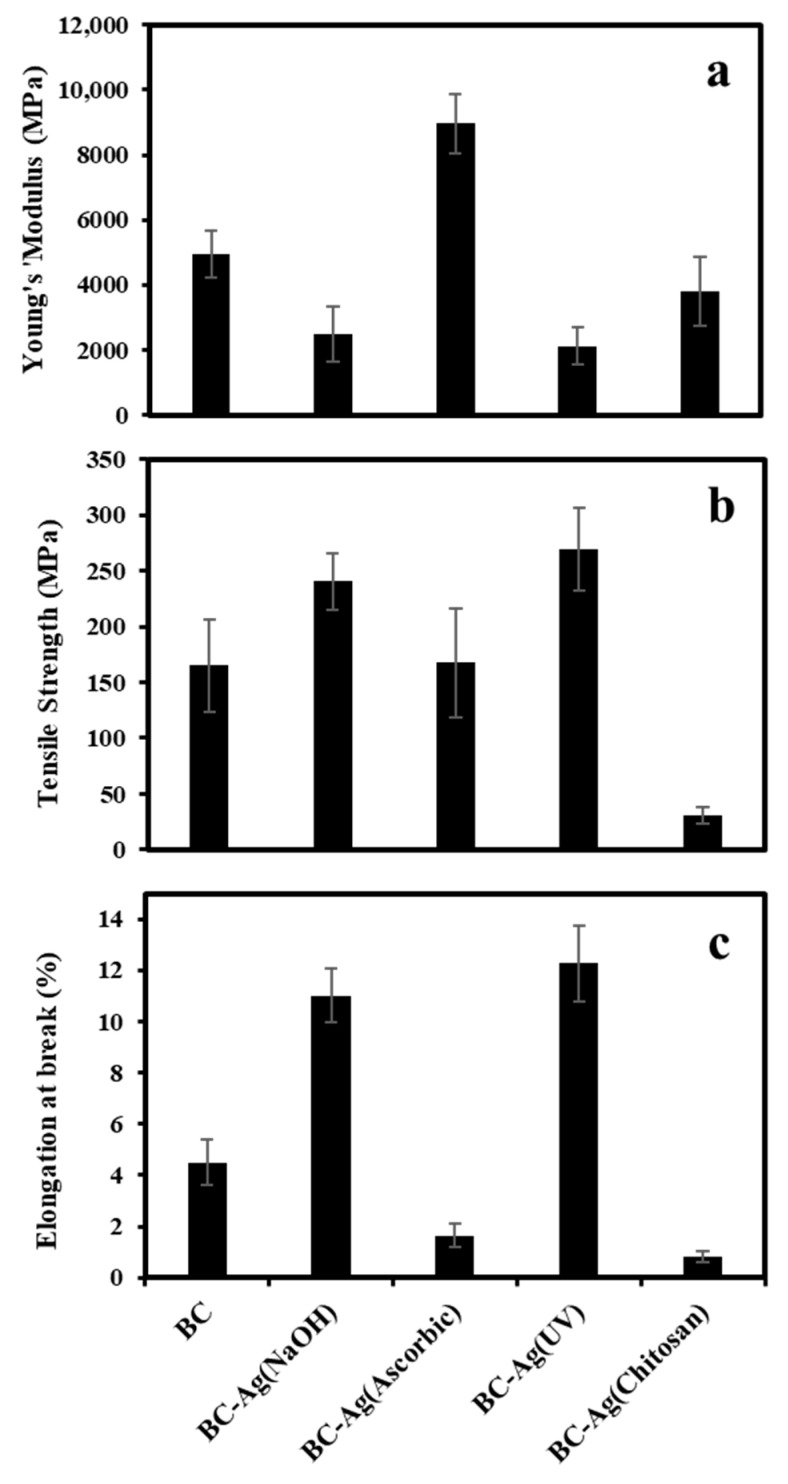
Young’s modulus (**a**), tensile strength (**b**), and elongation at break (**c**) of BC and BC-Ag composites.

The mechanical properties of BC and BC-Ag composites were investigated using a Universal Testing Machine to determine Young’s modulus, tensile strength, and elongation at break. The results of the testing are presented in Figure 6. The Young’s modulus, tensile strength, and elongation at break of the pristine BC film were measured to be 4945 M7Pa, 165 MPa, and 4.5%, respectively. 

The mechanical properties of BC-Ag composites varied greatly by the method of reduction. For BC-Ag(NaOH) and BC-Ag(UV) composites, Young’s modulus of the composites dropped dramatically by approximately 50%. Similarly, BC-Ag(Chitosan) exhibited a significant decrease in Young’s modulus by about 23%. On the other hand, the composites of BC-Ag(Ascorbic) demonstrated a remarkable increase in Young’s modulus, reaching 8960 MPa, which was almost twice the value of the pristine BC. These findings are consistent with the crystallinity values obtained from XRD analysis. Except for BC-Ag(Ascorbic), which did not contain Ag_2_O, other BC-Ag composites showed lower crystallinity, which might affect the mechanical properties of the films. The increase in Young’s modulus of BC-Ag(Ascorbic) could be partly attributed to the enhanced crystallinity, which has been known to increase Young’s modulus in polymers [45]. However, while BC-Ag(Chitosan) exhibited a lower crystallinity than BC-Ag(NaOH) and BC-Ag(UV), its Young’s modulus was relatively higher than in those composites. The effect of chitosan inside the matrix on the mechanical properties of BC-Chitosan has been previously investigated [28]. The composites of BC-Chitosan were prepared by immersing BC in chitosan solution. Tensile strength, elongation at break, and Young’s modulus of the BC-Chitosan films were found slightly lower compared to those of the BC films, and the crystallinity was decreased. It was suggested that the incorporation of chitosan into the BC pellicle might increase the amorphous phase fraction, which could weaken the affinity of the binding of the films. The changes in crystallinity and mechanical properties of BC-Ag(Chitosan) should therefore be affected by the incorporation of AgNPs into the polymer matrix and the presence of chitosan in the composite. 

BC-Ag(NaOH) and BC-Ag(UV) composites demonstrated a moderate increase in both tensile strength and elongation at break as compared to BC. Alternatively, BC-Ag(Ascorbic) showed a significant increase in Young’s modulus, but elongation at break of the film was decreased by about 60%, while the tensile modulus remained relatively unchanged. The significant decrease in elongation at break for BC-Ag(Ascorbic) could be attributed to the higher rigidity and stiffness of the materials, which resulted from the inclusion of highly crystalline silver nanoparticles. In the case of BC-Ag(Chitosan), the presence of chitosan inside the matrix could cause a reduction in flexibility, making the composites noticeably more brittle and resulting in a substantial decrease of approximately 80% in both tensile strength and elongation at the break as compared to BC. 

### 3.6. Thermal Properties 

**Figure 7:**
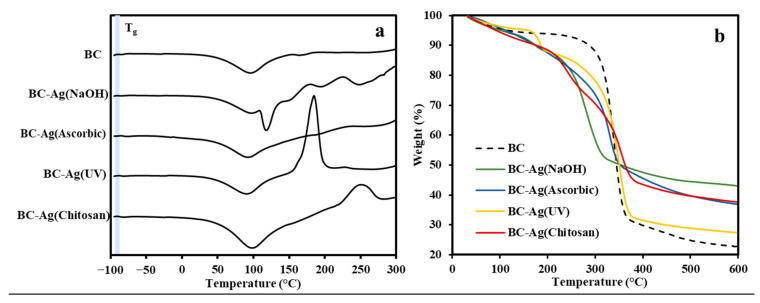
DSC thermograms (**a**) and TGA curves (**b**) of BC and BC-Ag composites.

The thermal properties of BC and BC-Ag composites were investigated using differential scanning calorimetry (DSC) (Figure 7a) and thermogravimetric analysis (TGA) (Figure 7b). The TGA curve illustrates the thermal degradation profiles of BC and BC-Ag composites, with the data tabulated in Table 1. For pristine BC, the results reveal several stages of thermal degradation. The first stage of minor weight loss occurred between room temperature and 150 °C, which can be attributed to the evaporation of residual water in the BC matrix [46]. The second stage, leading to significant weight loss, occurred between 280 °C and 380 °C and is attributed to cellulose degradation [47]. For the last stage, a small weight loss is observed between 400 °C and 600 °C, corresponding to the degradation of carbonaceous residues [46,48]. The degradation profiles of BC-Ag composites were relatively similar to those of BC, with additional weight loss observed around 180 °C to 240 °C for BC-Ag composites, which could potentially be associated with the decomposition of Ag_2_O and the capping layers of AgNPs. Additionally, BC-Ag(Chitosan) composite exhibited an additional weight loss at around 240 °C, likely due to chitosan decomposition [49]. The residue mass at 600 °C from TGA analysis can be used to estimate the silver content within the BC-Ag composite, with BC-Ag(NaOH) yielding the highest silver content, while BC-Ag(UV) yields the lowest silver content.

The glass transition temperature (T_g_) was determined using DSC thermograms; the obtained T_g_ data are tabulated in Table 1. The corresponding DSC curves are illustrated in Figure 7a. All samples exhibited an endothermic peak between 90 °C and 98 °C, which relates to the evaporation of water in the BC and BC-Ag composites. Additional endothermic peaks were observed in all BC-Ag composites, which, as mentioned earlier, could be attributed to the decomposition of Ag_2_O and the capping layers of AgNPs, as well as the decomposition of chitosan in the case of BC-Ag(Chitosan). The T_g_ values obtained from DSC were relatively similar across all samples, with BC-Ag composites having slightly lower or similar T_g_ values than BC, except for BC-Ag(Ascorbic), which was 4.8 °C higher than pristine BC. This result is in accordance with crystallinity index (CI) values obtained from XRD analysis, where BC-Ag composites typically exhibit lower CI, except for BC-Ag(Ascorbic).

### 3.7. Electrical Properties

**Figure 8:**
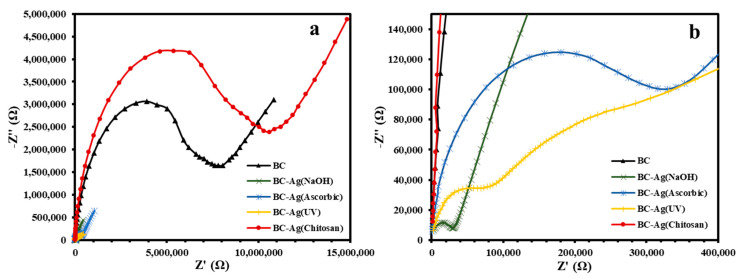
Electrochemical impedance spectroscopy (EIS) spectra (Nyquist plots) with the frequency range from 200 kHz to 1 Hz (**a**) and a magnified view of the high-frequency region of impedance spectra (**b**) for BC and BC-Ag composites.

An electrochemical impedance spectroscopy (EIS) study was carried out in a frequency range from 200 kHz to 1 Hz to measure the electrical properties of BC and BC-Ag composites. Figure 8 shows the Nyquist plots of BC and BC-Ag composites. The plots exhibited significant variations depending on the reduction method, with a smaller region of plotted semicircles indicating a higher electrical conductivity. The larger semicircle region observed in BC-Ag(Chitosan) compared to pristine BC could be partially attributed to the increased film thickness, resulting in higher electrical resistance. The estimated values of electrical conductivity for BC, BC-Ag(NaOH), BC-Ag(Ascorbic), BC-Ag(UV), and BC-Ag(Chitosan) were calculated from the semicircle region, resulting in calculated conductivities of 2.4 × 10^−10^, 1.1 × 10^−7^, 5.6 × 10^−9^, 1.8 × 10^−8^, and 5.8 × 10^−10^ S/cm, respectively. BC-Ag(NaOH) exhibited the highest conductivity, which was approximately three orders of magnitude higher than pristine BC. The highest conductivity from BC-Ag(NaOH) could be attributed to the high silver content within the matrix. This is supported by the highest residue observed at 600 °C in the TGA analysis (Table 1), while the lowest conductivity of BC-Ag(Chitosan) can be attributed to the non-conductive nature of chitosan within the composites. The presence of a double semicircle region in the Nyquist plot of BC-Ag(UV) suggests an uneven distribution of Ag within the composites, which could be due to UV light being unable to reach the inside of the cellulose matrix and only capable of reducing Ag ions on the outer part. 

### 3.8. Water Absorption Capacity

**Figure 9:**
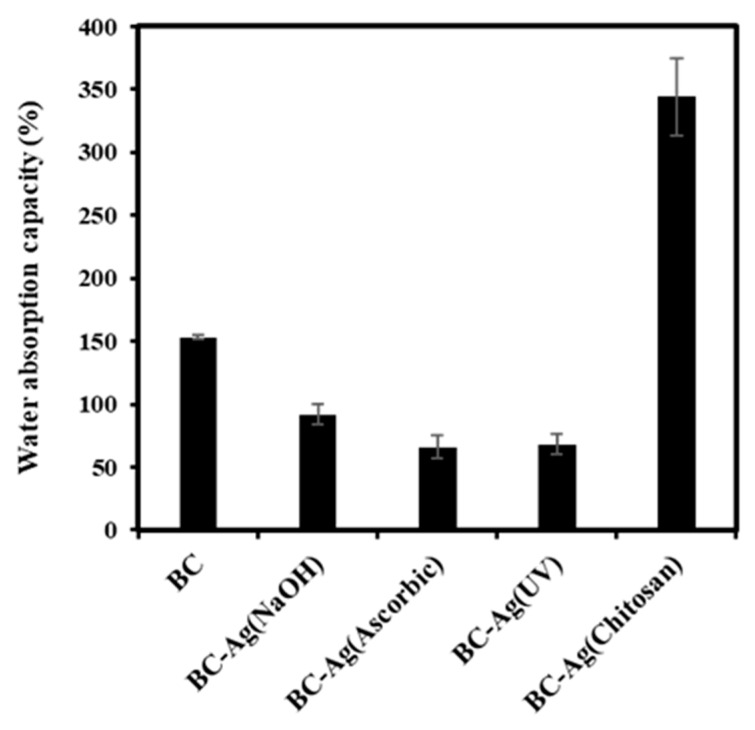
Water absorption capacity (WAC) of BC and BC-Ag composites.

The water absorption capacity (WAC) values of BC and BC-Ag composites were determined using dried films, and the results are illustrated in Figure 9. The WAC of BC, BC-Ag(NaOH), BC-Ag(Ascorbic), BC-Ag(UV), and BC-Ag(Chitosan) composites was 153%, 92%, 66%, 68%, and 344%, respectively. The high WAC of BC can be attributed to its porosity and surface areas. The water molecules are physically trapped both on the surface and within the BC matrix, which is composed of interconnected fibrils [50]. Additionally, hydrogen bonding facilitates the binding of water molecules with the BC fibrils [51]. 

Apart from BC-Ag(Chitosan), the other BC-Ag composites showed a lower WAC, which could be attributable to the incorporation of AgNPs into the BC matrix, which made the BC structure denser with lower porosity, thereby reducing water penetration and the WAC of the films. In contrast, the increase in the WAC values for BC-Ag(Chitosan) could be attributed to the highly hydrophilic nature of chitosan, which exhibited simultaneous interaction with both water molecules and BC chains [15], resulting in increased absorption of water molecules in the BC matrix, even with a lower pore size and a denser structure compared to BC [15]. 

### 3.9. Antibacterial Activity

**Figure 10:**
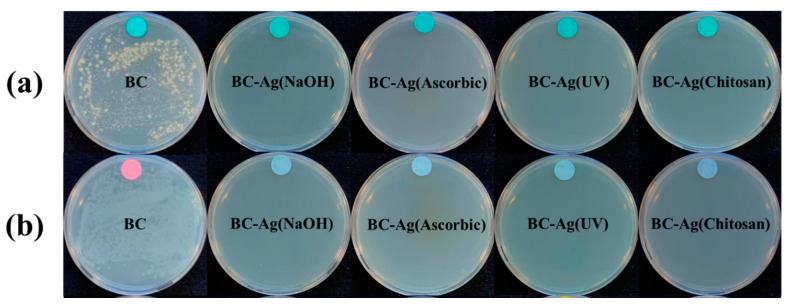
Antibacterial activity of BC and BC-Ag composites against *S. aureus* (**a**) and *E. coli* (**b**) investigated by the plate count method. A complete inhibition effect of BC-Ag composites on bacteria growth was observed.

BC and BC-Ag composites were tested for antibacterial activities against *S. aureus* and *E. coli*, which served as models of Gram-positive bacteria and Gram-negative bacteria, respectively. The test results are presented in Table 2. For pristine BC, there was a significant increase of 198% and 646% in the viable counts of *S. aureus* and *E. coli*, respectively. In contrast, the growth of *S. aureus* and *E. coli* was strongly inhibited by all types of BC-Ag composites (reduction from ~3 × 10^6^ CFU/mL to 0 CFU/mL), to the extent that no visible colonies were observed or detected on the agar plates (Figure 10). These results clearly demonstrated the antibacterial potential of AgNPs and indicated that the antibacterial properties originate from the presence of AgNPs rather than BC. Furthermore, the findings suggest that the antimicrobial effect of BC-Ag composites was not significantly influenced by the method of Ag reduction under the conditions in this study. However, it was previously reported that smaller AgNPs tended to exhibit higher antibacterial activity than larger AgNPs [43], due to the higher total surface area of the nanoparticles [52]. Silver particles were shown to strongly adhere to BC, preventing the leakage of AgNPs from the film [22,53]. When compared to other silver composites, the antibacterial efficiency of BC-AgNPs composites is similar to or even superior to that of some others. From the results obtained by Xie et al. [54], a chitosan hydrogel reinforced by silver nanoparticles with 39% Ag concentrations was developed, which exhibited 99.94% and 99.86% inhibition against *S. aureus* and *E. coli*, respectively. The antibacterial properties of BC-Ag composites can be primarily attributed to their ability to release silver ions (Ag^+^) upon contact with moisture [55], which interact with the bacterial cell wall and membrane, disrupting their structure and increasing cell permeability [56]. 

### 3.10. Advantages and Disadvantages of Each Reduction Method

Different reduction methods for synthesizing BC-Ag composites exhibit distinct advantages and disadvantages, as summarized in Table 3. NaOH reduction offers high silver content, conductivity, tensile strength, and elongation at break. However, it exhibits a low Young’s modulus. Ascorbic acid reduction results in the highest Young’s modulus, crystallinity, and increased thermal stability but leads to a larger AgNPs size and low elongation at break. UV irradiation reduction requires no additional chemical reagents and provides high tensile strength and elongation at break but has the lowest silver content and Young’s modulus. Chitosan reduction produces AgNPs with the smallest size and offers the highest water-absorption capacity. Moreover, the addition of chitosan to the composite could potentially enhance antibacterial and wound-healing properties due to its ability to release chitosan [28,57]. However, it exhibits low conductivity and crystallinity as well as low tensile strength and elongation at break, making it brittle. All reduction methods in this study exhibit high antibacterial properties against *S. aureus* and *E. coli*.

## 4. Conclusions

In this study, BC-Ag composites were successfully synthesized using various in situ reduction methods to convert Ag ions to AgNPs by using sodium hydroxide, ascorbic acid, chitosan, and UV irradiation. The effect of the reduction methods on various properties was investigated. It was observed that AgNPs were effectively attached to BC fibers. BC-Ag(Ascorbic) exhibited the largest size of AgNPs dispersed in the BC matrix, whereas those prepared by using chitosan displayed AgNPs of the smallest size. The result from X-ray diffraction analysis revealed that the incorporation of AgNPs reduced the crystallinity of the composite films, except for the BC-Ag(Ascorbic), which exhibited an increase in crystallinity. The mechanical properties of the BC-Ag composites varied greatly depending on the reduction methods. BC-Ag(Ascorbic) showed a remarkable increase in Young’s modulus. The composites of BC-Ag(NaOH) and BC-Ag(UV) showed a significant increase in tensile strength and elongation at break, whereas the BC-Ag(Chitosan) composite became brittle and exhibited a significant decrease in tensile strength and elongation at break. Thermal analysis indicated that the decomposition profiles and T_g_ of the composites were quite similar to those of BC; only a small increase in T_g_ was noticed for BC-Ag(Ascorbic). Additional weight-loss steps were observed for BC-Ag composites at high temperatures during TGA analysis. Electrochemical impedance spectroscopy analysis demonstrated that BC-Ag(NaOH) exhibited the highest conductivity at 1.1 × 10^−7^ S/cm, which was approximately three orders of magnitude higher than for pristine BC, while the conductivity of the other BC-Ag composites, except for BC-Ag(Chitosan), increased to some extent. The WACs of BC-Ag composites tended to decrease by the incorporation of AgNPs; however, the WAC of BC-Ag(Chitosan) increased owing to the presence of chitosan within the BC matrix. Antibacterial tests showed that all BC-Ag composites possessed a very strong inhibitory effect against *E. coli* and *S. aureus*. These multifaceted properties of BC-Ag composites highlight their potential applications in various fields such as antimicrobial packaging, wound dressings, and biomedical devices. These findings should contribute to a better understanding of the BC-Ag composites and open avenues for further exploration and optimization of their properties for specific applications.

## Figures and Tables

**Table 1:** Glass transition temperature (T_g_), maximum mass loss rate temperature (T_max_), mass loss (%), and residue at 600 °C of BC and BC-Ag composites.

Samples	T_g_ (°C)	1st Decomposition		2nd Decomposition		3rd Decomposition		Residue at 600 °C (%)
		T_max_ (°C)	Δ Mass loss (%)	T_max_ (°C)	Δ Mass loss (%)	T_max_ (°C)	Δ Mass loss (%)	
BC	−86.2	44.2	6.2	339.6	64.0	465.2	7.0	22.8
BC-Ag(NaOH)	−87.9	44.2	5.9	172.9	5.6	280.3	45.1	43.0
BC-Ag(Ascorbic)	−81.4	59.3	4.3	328.8	58.7	-	-	36.9
BC-Ag(UV)	−86.1	73.2	4.6	182.5	8.2	350.6	59.8	27.4
BC-Ag(Chitosan)	−86.1	65.5	8.9	241.4	17.6	354.4	35.8	37.6

Note: T_max_ was obtained from derivative thermogravimetric analysis (DTG); T_g_ was determined using differential scanning calorimetry (DSC) thermograms.

**Table 2:** Colony-forming unit counts (CFU/mL) at 0 h and 24 h contact time intervals with BC and BC-Ag composites against *S. aureus* (A) and *E. coli* (B).

		BC	BC-Ag (NaOH)	BC-Ag (Ascorbic)	BC-Ag (UV)	BC-Ag (Chitosan)
(A)	0 h	2.59 × 10^6^	3.21 × 10^6^	3.29 × 10^6^	3.30 × 10^6^	3.23 × 10^6^
	24 h	7.72 × 10^6^	0	0	0	0
	Reduction (Log CFU/mL)	−7.41%	100%	100%	100%	100%
(B)	0 h	1.76 × 10^6^	3.53 × 10^6^	3.55 × 10^6^	3.57 × 10^6^	3.52 × 10^6^
	24 h	1.32 × 10^7^	0	0	0	0
	Reduction (Log CFU/mL)	−13.99%	100%	100%	100%	100%

Note: Negative (−%) values of reduction indicate an increase in viable counts of bacteria.

**Table 3:** Summary of advantages and disadvantages of each silver reduction method.

Methods of Reduction	Advantages	Disadvantages
NaOH reduction	High silver content High conductivity High tensile strength and elongation at break High antibacterial activities	Low Young’s modulus
Ascorbic acid reduction	High Young’s modulus High crystallinity Increased thermal stability High antibacterial activities	Large AgNPs size Low elongation at break
UV irradiation reduction	No chemical reagents required High tensile strength and elongation at break High antibacterial activities	Low silver content Low Young’s modulus
Chitosan reduction	Small AgNPs size High water absorption capacity High antibacterial activities and improved wound healing properties due to chitosan [28,57]	Low conductivity Low tensile strength and elongation at break (brittle) Low crystallinity

## Data Availability

The data supporting reported results of this study are available on request from the corresponding author.
